# Identifying opportunities to support patient-centred care for ductal carcinoma in situ: qualitative interviews with clinicians

**DOI:** 10.1186/s12885-020-06821-5

**Published:** 2020-04-30

**Authors:** Bryanna B. Nyhof, Frances C. Wright, Nicole J. Look Hong, Gary Groot, Lucy Helyer, Pamela Meiers, May Lynn Quan, Nancy N. Baxter, Robin Urquhart, Rebecca Warburton, Anna R. Gagliardi

**Affiliations:** 1 Toronto General Hospital Research Institute, University Health Network, 200 Elizabeth Street, Toronto, Ontario M5G2C4 Canada; 2 Sunnybrook Health Sciences Centre, Toronto, Ontario Canada; 3 University of Saskatchewan, Saskatoon, Saskatchewan Canada; 4 Dalhousie University, Halifax, Canada; 5 University of Calgary, Calgary, Canada; 6 St Michael’s Hospital Department of Surgery and Li Ka Shing Knowledge Institute, Department of Surgery and the Institute for Health Policy Management and Evaluation, Toronto, Canada; 7 University of Toronto, Toronto, Canada; 8 University of British Columbia, Vancouver, Canada

**Keywords:** Ductal carcinoma in situ, Patient-centred care, Communication, Decision-making

## Abstract

**Background:**

Women with ductal carcinoma in situ (DCIS) report poor patient-clinician communication, and long-lasting confusion and anxiety about their treatment and prognosis. Research shows that patient-centred care (PCC) improves patient experience and outcomes. Little is known about the clinician experience of delivering PCC for DCIS. This study characterized communication challenges faced by clinicians, and interventions they need to improve PCC for DCIS.

**Methods:**

Purposive and snowball sampling were used to recruit Canadian clinicians by specialty, gender, years of experience, setting, and geographic location. Qualitative interviews were conducted by telephone. Data were analyzed using constant comparison. Findings were mapped to a cancer-specific, comprehensive PCC framework to identify opportunities for improvement.

**Results:**

Clinicians described approaches they used to address the PCC domains of fostering a healing relationship, exchanging information, and addressing emotions, but do not appear to be addressing the domains of managing uncertainty, involving women in making decisions, or enabling self-management. However, many clinicians described challenges or variable practices for all PCC domains but fostering a healing relationship. Clinicians vary in describing DCIS as cancer based on personal beliefs. When exchanging information, most find it difficult to justify treatment while assuring women of a good prognosis, and feel frustrated when women remain confused despite their efforts to explain it. While they recognize confusion and anxiety among women, clinicians said that patient navigators, social workers, support groups and high-quality information specific to DCIS are lacking. Despite these challenges, clinicians said they did not need or want communication interventions.

**Conclusions:**

Findings represent currently unmet opportunities by which to help clinicians enhance PCC for DCIS, and underscore the need for supplemental information and supportive care specific to DCIS. Future research is needed to develop and test communication interventions that improve PCC for DCIS. If effective and widely implemented, this may contribute to improved care experiences and outcomes for women diagnosed with and treated for DCIS.

## Background

Ductal carcinoma in situ (DCIS), which comprises up to 25% of all screen-detected abnormalities, includes a spectrum of abnormal cell types confined to the breast ducts with variable natural history, and risk for progression and recurrence [1–4]. Because there is no test to establish whether DCIS will progress to invasive breast cancer, recommended treatment for DCIS is similar to invasive breast cancer and includes surgery (lumpectomy or mastectomy) with or without adjuvant radiation or hormone therapy [5, 6]. Adequately treated DCIS has a favourable prognosis, with 20-year breast cancer specific mortality rate of 3.3% (95% CI 3.0 to 3.6) [7]. Therefore, the goal of treatment is to prevent progression to invasive breast cancer, or DCIS recurrence that may progress, while also conserving healthy breast tissue and minimizing unnecessary harm.

Despite the good prognosis, women with DCIS have long-lasting confusion and anxiety about their diagnosis and treatment [8–10]. For example, in a cross-sectional survey of 144 women diagnosed with DCIS in Australia, many were unsatisfied with information provided and reported misperceptions of risk; 60.0% incorrectly thought DCIS would metastasize, only 19.0% knew that not all women with untreated DCIS would develop invasive cancer, and 43.0% worried about dying from their disease [11]. Similarly, research found that women with DCIS have comparable concerns about dying from breast cancer and experience similar psychosocial distress as women with invasive breast cancer [12].

In contrast to the numerous studies exploring the experiences of women with DCIS, we found only three studies that examined how physicians approach DCIS discussions. When surveyed, 22% of 296 physicians in England, and 78% of 151 physicians in the United States reported difficulty explaining DCIS and treatment options to women [13, 14]. Both studies also found that communication practices varied across physicians including whether DCIS was described as cancer, a particularly confusing issue for women contributing to psychosocial distress. More recently, a mixed-methods study found that variation in DCIS terminology among surgeons was influenced by seniority and geographical region within England [15]. These studies were conducted a decade ago and may not reflect current views or practices, findings may not apply beyond the United States and England, and survey data did not fully explore the challenges that physicians experienced or their views on how to improve DCIS discussions.

Patient-centred care (PCC) offers a promising approach for optimizing patient health care experiences and outcomes. PCC is defined as care that informs, educates, engages and activates patients and their family/care partners consistent with their clinical needs, life circumstances, and personal preferences [16]. Considerable research shows that PCC has enhanced multiple patient outcomes across numerous conditions and settings including improved communication and relationship with providers, service experience, knowledge, and quality of life, and reduced anxiety, missed work, readmission rates, and mortality [17–19]. For example, a randomized controlled trial of communication training for both cancer patients and physicians increased patient engagement, and discussion of emotions, prognosis, and treatment choices (0.34, 95% CI 0.06–0.62, *p* = 0.02) [20].

PCC is widely advocated [21, 22], and could support and improve patient-clinician communication about DCIS. Our recent scoping review of research published from 1997 to 2016 on DCIS communication found no studies that implemented or evaluated interventions to facilitate PCC for DCIS [23]. Hence, research is needed to generate detailed insight that would inform intervention development. The purpose of this study was to interview clinicians who care for women with DCIS, and characterize challenges that influence DCIS communication and the type of interventions needed to facilitate patient-centred discussions. By identifying patient-centred approaches to support women and/or clinicians that could be broadly implemented, ultimately this may improve patient care experiences among women with DCIS, and psychosocial outcomes among DCIS survivors.

## Methods

### Approach

To gather detailed insight on little-known clinician views about DCIS communication challenges and supports, we employed a qualitative research design [24]. Specifically, we used a basic descriptive qualitative approach [25]. This method does not aim to generate theory; instead, it elicits straightforward descriptions in the participants’ own words about their views and experiences [26]. We conducted telephone interviews, which allowed for convenient and flexible scheduling with busy clinicians from various geographical locations across Canada. Rigour was ensured by complying with the 32-item Consolidated Criteria for Reporting Qualitative Research checklist [27]. We further enhanced rigour by sampling participants with various characteristics, using open-ended questions, exploring responses inductively, and independently deriving and comparing themes [28, 29]. This study was approved by the University Health Network research ethics board. All participants provided written informed consent prior to the interview. The interviewer had no relationship with participants.

### Sampling and recruitment

Eligible participants were practicing, English-speaking clinicians who care for women with DCIS including general surgeons, surgical, radiation and medical oncologists, radiologists, registered nurses and patient navigators. Clinicians were identified using publicly available databases and invited to participate by regular mail. Sampling of surgeons and oncologists was purposive by specialty, geographical location, hospital setting (academic, community), years of experience and gender. As qualitative research collects detailed data from representative participants, we aimed to interview a minimum of 5 clinicians of each aforementioned specialty who varied in non-mutually exclusive fashion in other sampling characteristics, for a minimum total of 30 participants, a common target in qualitative research, which unlike quantitative research, seeks to gather detailed information from a comparatively small number of participants. Ultimately, sample size in qualitative research is determined by informational saturation, meaning new themes do not emerge with successive interviews, which was assessed through discussion among the research team. We mailed 891 recruitment packages on March 10, 2017 and reminder packages on May 19, 2017. Snowball sampling, whereby interviewed participants referred us to other potential participants, [30] was used to recruit radiologists, breast cancer nurses and patient navigators. We contacted individuals who returned signed consent forms to schedule an interview.

### Data collection

BBN conducted telephone interviews between April 25, 2017 and August 20, 2017. BBN conducted telephone interviews with consenting participants between April 25, 2017 and August 20, 2017. Interview questions were informed by elements that comprise PCC such as patient-provider communication and patient involvement in decision-making, but kept purposefully broad and open-ended to avoid leading or biasing responses [31]. The interview guide was reviewed by all research team members, which included 7 breast cancer surgeons, prior to first use and again after review of the first 4 interview transcripts to improve wording and flow of questions. At the outset of each interview, clinicians were informed that the purpose was to understand how to achieve PCC for DCIS. PCC was defined to them as care that informs, educates, engages and activates patients consistent with their needs and values [16]. Clinicians were then asked about their approach for communicating with women about DCIS diagnosis, treatment and follow-up; barriers of communication; and recommendations for strategies, interventions or tools that could improve PCC for DCIS.

### Data analysis

BBN and ARG independently analyzed the first four transcripts, and compared and discussed analysis to jointly generate a preliminary codebook of themes and exemplar quotes. This was shared with the research team, who provided feedback on the codebook. With the guidance of ARG, BBN analyzed all remaining transcripts concurrent with data collection using constant comparison [32]. First, we extracted and categorized all data from each transcript by theme (level one), then merged and separated themes (level two) [33]. Quotes were organized by theme, and clinician specialty and province to identify similarities and differences. The research team reviewed themes and exemplar quotes on two occasions and provided feedback.

To generate a thorough understanding of how DCIS discussions could be improved, we mapped themes to a PCC framework developed by McCormack et al. comprised of 6 domains: 1) facilitating healing relationships, 2) exchanging information, 3) responding to patients’ emotions, 4) managing uncertainty, 5) making decisions, and 6) enabling patient self-management [34]. We chose the McCormack PCC framework because it was rigorously developed; informed by multiple rounds of input from patients and clinicians, and thus represents what patients and clinician consider ideal; more comprehensive of the components of PCC compared with several other PCC frameworks [35, 36], and specific to cancer unlike other PCC frameworks. Thus, it represents an ideal against which to compare clinician-reported DCIS discussion practices and challenges. Mapping of communication practices and challenges to the framework identified whether and how clinicians addressed PCC, thereby identifying specific gaps or inconsistences, which represent opportunities for improving PCC for DCIS. .

## Results

### Participants

Of the 891 clinicians invited to participate, 838 did not respond. Among the 53 that responded, 17 were not eligible. We obtained consent from 36 clinicians. An additional 12 clinicians recruited via snowball sampling consented to participate in this study. Of the 48 clinicians that consented, we were unable to schedule an interview with 2. In total, 46 clinicians were interviewed (Table 1).

**Table 1:** Participant characteristics

Characteristic	n (% of 46)
Specialty	
General Surgeon	9 (19.5)
Surgical Oncologist	10 (21.7)
Radiation Oncologist	12 (26.1)
Medical Oncologist	4 (8.7)
Radiologist	5 (10.8)
Registered nurse/ patient navigator	6 (13.0)
Province	
British Columbia	12 (26.1)
Alberta	2 (4.3)
Manitoba	3 (6.5)
Ontario	21 (45.6)
Nova Scotia	2 (4.3)
Newfoundland	6 (13.0)
Hospital Setting	
Academic	39 (84.7)
Community	7 (15.2)
Self-reported years of practice	
< 10 years	28 (60.8)
≥ 10 years	18 (39.1)

### Themes by PCC domain

Data organized by theme and participant characteristics is available in Additional file 1 Themes are described here by PCC domain, [34] and summarized along with exemplar quotes in Table 2.

**Table 2:** Themes and exemplar quotes by PCC domain [34]

PCC domains	Themes	Exemplar quotes
Fostering healing relationship	Building rapport	You have to you know take the time to get to know the patient well enough to be able to tailor your approach to them. (06 gen surg)
		You have to have the ability as a physician to say, you have all the time you need and I’m here, I always sit down with the patient and give them the correct idea that I’m listening, that I do have time. (05 surg onc)
Exchanging information	Label for DCIS	Some people talk about DCIS as being a pre-cancer and patients often come in from the surgeons saying that, but I don’t agree with that particular term. So, if that’s the case I clarify that it is breast cancer, but it’s very early (04 rad onc)
	Achieving patient understanding	The main barrier honestly for the most part is a patient just having the capacity to understand the notion of a non-invasive breast cancer and the potential for it to develop into an invasive breast cancer and that it’s not currently a threat; patients have a hard time wrapping their head around that notion. (07 rad onc)
		I see my patients at 6-month follow-up following surgery … and when I see them again, sometimes patients are still asking me whether or not they actually had cancer or whether it was DCIS or not. (04 gen sx)
		You end up spending a lot of time explaining to them the difference between DCIS and invasive breast cancer because they don’t necessarily understand that often when they come. They know they have breast cancer and that’s all they understand. (04 med onc)
	Justifying treatment despite good prognosis	They find it harder to understand why they need a mastectomy for pre-invasive disease … it’s not that serious but you need a mastectomy. That can be a difficult discussion (03 surg onc)
	Providing supplemental information	I have my own personal website which has a whole bunch of websites on it. So I kind of refer them to that to look at if they’re of that … wanting more information. So like I said, I have a personal website. It has lists of places you can go. Like the Cancer Society and the NIH and places they can search for reputable information (01 gen surg)
		I refer patients to information from randomized trials because with DCIS there are many randomized trials; and very consistent information from the randomized trials. So I like to use that information (04 rad onc)
		If they want more information … I’m not sure where or who I’d send them too? if they have questions along those lines, like someone else ends up dealing with them more than me. (10 surg onc)
		I’ll often times draw a sketch or show diagrammatically you know how … how for instance DCIS hasn’t invaded through base membrane. So I draw a picture of what a milk duct looks like and show how cancer cells populate and multiply. (05 gen surg)
Addressing Emotions	Emphasizing good prognosis	I usually try to explain to the patients that it represents the best form of breast cancer if they were ever going to get problem of this nature. (05 gen surg)
	Referring to supportive care	These are high maintenance emotional needs patients. So having somebody that they can access after the consultation is over just alleviates a lot of their psychological consternation. (09 surg onc)
Managing Uncertainty	Describing uncertainty	I tell them that not all ductal carcinoma in situ will progress to cancer. We don’t know exactly which ones will and which ones won’t. (03 gen surg) The surgery is more as a precaution to prevent further development of invasive disease but more also to make sure that that’s all that there is there. (07 surg onc)
Making Decisions	Involving women in decision making	I make sure I know before I go in with the patient whether or not it is amenable to do breast conserving surgery. I’ll make sure they have no contraindications of radiation … then I can present to them the options. But I don’t want to present options that are inappropriate. Then it’s at the patient choice, which they prefer. (08 surg onc)
		Some women choose to have a mastectomy for this condition but I certainly make it very clear that for that woman that is not necessary and it could be completely addressed without a mastectomy with a much lesser surgery, less invasive, fewer complications, etc., and then I would encourage that. (09 surg onc)
		If the area looks readily resectable I don’t even mention the word mastectomy. I just say we’re gonna, we’ll get this area out. (04 surg onc)
Enabling Self-Management	Paucity of DCIS-specific resources and support	I don’t think there’s anything that’s specific to DCIS. A lot of it is kind of it is around the surgery and what to expect at the time of surgery and if they need a wire localization procedure. What that involves and that kind of stuff.. (02 surg onc)
		I don’t think there are ones [support groups] for DCIS specifically. I think they’re all breast … that’s the problem, breast cancer patients. So these people are going to support groups with cancer people which is not ideally the best way. (07 surg onc)

#### Fostering a healing relationship

##### Building rapport

Clinicians said that, to achieve PCC, they established rapport with women by exhibiting patience and ensuring women know they are listening, and by exchanging pleasantries as a way of getting to know the patient. To convey these characteristics, clinicians said they sat at the same level, directly across or beside patients, and made eye contact throughout discussions. They also made a point of asking women questions about themselves before discussing clinical issues.

#### Exchanging information

##### Label for DCIS

Clinicians varied in whether they described DCIS as a form of cancer. Terms used appeared to be influenced by personal beliefs about whether DCIS was distinct from, or a precursor to invasive breast cancer. Many clinicians considered the potentially benign behaviour of DCIS to be uniquely different than invasive breast cancer and described DCIS using terms such as ‘abnormal cells’ or ‘something between normal and cancer’. Clinicians who themselves viewed DCIS as distinct from cancer said they spent time with women to eliminate confusion by distinguishing DCIS from invasive carcinoma. They also purposely avoided using the word ‘cancer’ when describing DCIS. Alternatively, several clinicians believed that, although DCIS is contained within the breast ducts, it is still a form of cancer, and employed terms such as ‘early stage breast cancer’ and ‘stage-0 breast cancer’ to describe DCIS.

##### Achieving patient understanding

Clinicians said they invested considerable time in explaining DCIS, sometimes over multiple appointments, but many patients remained confused about their diagnosis even after treatment, which was a source of frustration for clinicians.

##### Justifying treatment despite good prognosis

Clinicians said it was challenging for them to justify treatment, particularly when mastectomy was recommended, while at the same time assuring women of a good prognosis. They said this further added to confusion among women, and their own frustration.

##### Providing supplemental information

Many clinicians said they referred women to web sites to acquire supplemental information about their diagnosis and treatment; however, they said such resources were inconsistent in quality and readability. Others said they wanted to provide women with more information but were unaware of high quality, up-to-date Internet resources for DCIS. Most clinicians said that, in the absence of well-developed information or communication tools for DCIS, they used self-written or self-drawn notes or diagrams to facilitate discussions with women.

#### Recognizing and responding to emotions

##### Emphasizing good prognosis

While clinicians recognized that women with DCIS are often emotional and apprehensive during appointments, few described strategies for addressing emotions. Those who did said they attempted to alleviate concerns by emphasizing a good prognosis associated with DCIS.

##### Referring to supportive care

Instead of directly offering emotional support, a few clinicians referred women to patient navigators or social workers for further information and support. Doing so allowed them to focus limited time on explaining important clinical issues, knowing that others would address women’s emotions and concerns. However, most clinicians said they did not have access to navigators or social workers for women with DCIS, or referrals were prioritized for women with invasive breast cancer.

#### Managing uncertainty

##### Describing uncertainty

Few clinicians said they discussed the likelihood of untreated DCIS progressing to invasive cancer with patients. Those who did employed vague terms such as likely or unlikely. None employed statistics or scientific evidence as the basis for discussion.

#### Making decisions

##### Involving women in decision making

When asked about their approach to involving women in decision-making, clinicians instead described the treatment options they offered to women. Many said they first described all available treatment options with patients and then recommended one option. Others said they mentioned and recommended only one treatment option as a way of preventing women from choosing mastectomy based on anxiety or fear in instances where lumpectomy was appropriate.

#### Enabling self-management

##### Paucity of DCIS-specific resources and support

Clinicians said that despite wanting to help women access self-care advice or support, they were unaware of such resources. They emphasized that information tools such as pamphlets or web sites, or services offered by patient navigators or support group focused on invasive breast cancer or offered little information specific to DCIS, and were therefore not helpful for women with DCIS.

### Challenges or variability in PCC for DCIS

Clinicians described approaches they used to address the PCC domains of fostering a healing relationship, exchanging information, and addressing emotions. However, many clinicians described challenges or variable practices for all PCC domains but fostering a healing relationship (Table 3). Clinicians vary in describing DCIS as cancer. When exchanging information, most find it difficult to justify treatment while assuring women of a good prognosis, and feel frustrated when women remain confused despite their efforts to explain it. Even though they recognize confusion and anxiety, clinicians said that resources specific to DCIS that would address emotions such as patient navigators, social workers, support groups or high-quality information are lacking. Clinicians do not appear to be managing uncertainty, involving women in making decisions, or enabling self-management.

**Table 3:** Summary of challenges and variability in PCC for DCIS

PCC domain	Approaches	Challenges or Variability
Fostering Healing Relationships	• Exhibit patience	–
	• Affirm they are listening	
	• Exchange pleasantries	
	• Sit at same level	
	• Make eye contact	
Exchanging information	• Distinguish DCIS from invasive cancer	• Clinicians differed in whether they believed DCIS was cancer; as a result, terms used to describe DCIS also differed: ‘abnormal cells’ versus ‘early stage breast cancer’
	• Avoid using the word ‘cancer’	
	• Spend time explaining DCIS, sometimes over multiple appointments	• Frustrating when patients remain confused about DCIS despite their efforts to explain it
		• Difficult to justify treatment while also assuring women of a good prognosis; this was frustrating because they knew it further confused women
	• Use self-drawn notes or diagrams in additional to verbal information	
		• Internet information of poor quality; unaware of good quality Internet information on DCIS
	• Refer women to web sites for supplemental information	
Addressing Emotions	• Alleviate concerns by emphasizing a good prognosis	• Recognize that women with DCIS experience anxiety and concerns but do not directly address emotions
	• Refer women to patient navigators, social workers for information and emotional support	
		• Lack access to patient navigators or social workers
Managing Uncertainty	–	• Uncertainty not defined or explained in brief, vague terms (i.e. unlikely)
		• None shared statistics or scientific evidence
Making Decisions	–	• Describe options but recommend one
		• Describe and recommend only one option
Enabling Self-Management	–	• Self-care advice or support specific to invasive breast cancer
		• Unaware of DCIS-specific self-management resources

### Interventions to support PCC for DCIS

Despite the aforementioned challenges, when asked about interventions to support patient-centred discussions about DCIS, clinicians said they did not need or would not use training or communication tools because they believed their current approach to communication was sufficient.

I’m not sure how useful that would be for me specifically … I’ve got a pretty refined process that I probably even if something was really great and available I’d probably you know … not a tool I’d start using. Over 30-years evolved approach that I find usually works. So I probably wouldn’t personally find additional tools development helpful (01 rad onc).

I can’t think of where it would be helpful for me or haven’t had any patient’s articulate sort of a disappointment or a need. I don’t know, I don’t see patients as feeling as lost as invasive patients in terms of wishing they had the support group or. So I don’t know (08 surg onc).

I haven’t really had patients say, you know I really don’t know what I’m doing or what this is about … I don’t know that there’s a need for something extra specifically on communication around DCIS (03 surg onc).

## Discussion

By comparing self-reported communication practices against a cancer-specific, comprehensive PCC framework [34], this study identified that many clinicians refer to DCIS as cancer, and experience difficulty and frustration in describing DCIS and justifying the need for treatment in a way that is understood by patients. While they recognize that women experience confusion and anxiety, they are unable to offer supplemental help because information, patient navigators and support groups for DCIS are not available. Despite these issues, clinicians said they did not need or would not use communication tools or training to support or improve PCC for DCIS.

Clinicians in our study and elsewhere [13, 14] used inconsistent and conflicting terminology to describe DCIS. The present study provides additional insight regarding the basis of this variation; use of labels may depend on ones’ interpretation of DCIS as either unique from or similar to breast cancer. Referring to DCIS as cancer contributes to patient confusion, anxiety, and distress, leading to reduced health-related quality of life and undermining informed decision-making [11, 37, 38]. Others have advocated that more nuanced nomenclature for DCIS be developed [39]. Research is needed to explore the benefits and harms of various labels for DCIS through input from women and clinicians to optimize language for DCIS. Greater clarity and consistency in language used to describe DCIS may help clinicians when discussing DCIS and improve the patient experience.

Clinicians in our study who described communication challenges and lack of high-quality information tools said they would not benefit from DCIS communication tools.. Decision aids can support patients in shared decision-making by eliciting preferences about treatment options and associated outcomes, but analysis of 21 decision aids for women with early-stage breast cancer revealed that many failed to comply with widely-used decision aid standards; offer data on outcome probabilities, information essential to decision-making; or offer guidance on how to elicit patient preferences [40]. Furthermore, a cohort study found that most patients given a self-administered decision aid reviewed the information, but few made notes or answered questions as prompted by the decision aid, or shared that information with their doctors [41]. Shared decision-making constitutes only one of six domains in the PCC framework, which may explain the limited uptake. Further research is needed to understand what type of communication aid would better support patient-centred discussions about DCIS.

Participating clinicians also said they would not benefit from training to improve communication about DCIS. Research in both chronic conditions and oncology suggests that physician communication training can change behaviour and improve patient outcomes. For example, a randomized controlled trial found that training interventions targeted to both oncologists and cancer patients significantly improved patient-provider communication [20]. Similarly, a Cochrane systematic review found that PCC training for clinicians transferred and improved communication skills among clinicians, resulting in greater engagement of patients in discussions and decision-making [42]. In particular, the review found that short-term training (< 10 h) was as successful as lengthier training, which may encourage clinicians to participate [43]. Further research is needed to develop and evaluate training aimed at patients and/or clinicians that could structure and improve patient-clinician communication by more fully addressing PCC domains and sub-domains. A Cochrane systematic review of interventions to support PCC based on 43 randomized controlled trials found that educational material showed greater benefit in patient behaviour, satisfaction, and outcomes, and on the communication process when aimed at both patients and clinicians rather than targeting one group only, an approach that may overcome clinician reluctance by empowering patients to be better prepared for and take part in DCIS discussions [42].

Similar to previous research [11], clinicians in the present study reported that women with DCIS are often confused and would benefit from having access to patient navigators (PNs), however, PNs were not always available to women with DCIS. The role of PNs may provide patients and physicians with support to improve patient care experiences. The navigation role provides a measure of security and familiarity through continuity of care and integrates multiple areas of care coordination, including streamlining medical appointments, providing education and assisting with access to financial services and psychological support throughout and following treatment [44, 45]. In cancer care, research shows that patients who had access to PNs reported fewer problems with care and improved delivery of PCC [46, 47]. There are several barriers to implementation of PNs, including availability of trained PNs, distribution of PNs across rural or remote regions, and health care costs associated with use of PNs. Although PNs have traditionally been nurses, they can also be social workers, volunteers or previous patients [48]. Training for PNs is not yet regulated or required in some jurisdictions; however, PN training developed by the National Cancer Institute [49] was designed for individuals with no prior health care experience and can be delivered over a 3-day training period [48]. Thus, strategies are available to increase the number of trained PNs over a short training period. Because PNs may be scarce in rural or remote regions, future research should investigate the value of access to PNs by telephone or an online service for women with DCIS [50, 51].

This study was the first to thoroughly explore the communication practices and perceptions of clinicians who care for women with DCIS. We employed rigorous methods, [24, 25, 28, 29] and adhered to qualitative research reporting standards [27]. Other strengths include the diversity of participants (geographic region, specialty, years in practice, setting), and achieving informational saturation, which enhances transferability of the findings. Members of our research team included practicing clinicians who care for women with DCIS; they independently analyzed and interpreted study findings, offering critical, real-world feedback to enhance insight and recommendations. Some limitations of the present study must be noted. Although we invited many clinicians with various characteristics, a small proportion responded as is common in qualitative research; thus, there may be potential for responder bias, meaning that clinicians who were especially interested in DCIS or PCC agreed to be interviewed, which may have influenced responses. Despite our recruitment efforts, only 7 of 46 participating clinicians practiced in non-academic facilities, so the views and experiences of academic clinicians represented the majority of our findings. Given that all participants practiced in Canada, the findings may not be relevant elsewhere. The McCormack et al. PCC framework is not necessarily a “gold standard”, [34] but as the most comprehensive PCC framework reflecting the priorities of cancer patients, it offered a basis for further interpreting study findings. While this article did not compare or correlate clinician findings with the views of women with DCIS, we have conducted focus groups with DCIS survivors and will report that data elsewhere.

## Conclusion

By interviewing clinicians who care for women with DCIS and comparing their self-reported practices to a comprehensive, cancer-specific PCC framework, this study identified numerous frustrating communication challenges experienced by clinicians and variability in communication practices across clinicians. Although clinicians said they did not need or want communication interventions, the findings represent currently-unmet opportunities by which to help clinicians enhance PCC for DCIS including exchanging information, managing uncertainty, recognizing and responding to emotions, making decisions, and enabling self-management. Findings also underscore the need for supplemental information and supportive care specific to DCIS.

## Supplementary information

**Additional file 1.** Themes and exemplar quotes. Table of study data including themes and corresponding participant quotes

## Data Availability

All data generated or analysed during this study are included in this published article and its supplementary information file (Additional file 1.pdf) titled ‘complete clinician data set’, which is a table of all relevant themes and quotes from interviewed clinicians.

## Abbreviations

DCIS: Ductal carcinoma in situ
PCC: Patient-centred care
