# Supplementary material for: Identifying opportunities to support patient-centred care for ductal carcinoma in situ: qualitative interviews with clinicians
Source: BMC Cancer. 2020 Apr 30;20:364. doi: 10.1186/s12885-020-06821-5 (PMC7191683; doi:10.1186/s12885-020-06821-5)
Supplement: Supplementary file 1 — Additional file 1. Themes and exemplar quotes. Table of study data including themes and corresponding participant quotes [file 12885_2020_6821_MOESM1_ESM.docx]

Additional File 1. Themes and quotes

Fostering a healing relationship

| Theme | Clinician response |
| --- | --- |
| Building Rapport | Exhibits patience  there’s always the perception that I’m taking the time from the doctor, the doctor’s time is very important so I have to rush it, he doesn’t have too much time. And you have to…have the ability as a physician to say, well…you have all the time you need and I’m here, I always sit down with the patient and give them the correct idea that I’m listening, that I do have time. (05 surg onc)  Well I sit with them, so I give them the appearance that they have as much time as they need for once so that they don’t feel it’s left and then we go over it several times…then we precede the booking of surgery. (08 surg onc)  Personal enquiries  I think it’s possible to explain these things to most people in a fashion that you know that they can understand but you have to…you have to you know take the time to… get to know the patient well enough to be able to tailor your approach to them…it’s our job to understand who this woman is and to speak in a language that…she understands. It’s a problem but I don’t think it’s a…I don’t think it should present a barrier. I think it’s something that we should try to recognize and issue the…it should be something to present opportunities for us to use you know their particular language, a set of skills you know to explain to them you know more what the important and pertinent points of the disease are. (06 gen sx) |

| **Exchanging Information** | | Clinician response |
| --- | --- | --- |
| Issue | Theme |  |
| Label for DCIS | DCIS as unique from cancer/ pre-cancer | I usually talk about it as a continuum between kind of normal breast tissue and invasive breast cancer…I use pre-cancer as my kind of go too. I report to it as pre-cancer to my patients because otherwise that they hear cancer, I think they get a little bit more worried than what I think they maybe should (01 surg onc)  I try and explain what DCIS means and in plain language I usually describe it as pre-cancerous cells that if left alone could turn into a breast cancer; as sort of the general types of words that I use to explain it (02 surg onc)  when they come back I’ll you know go through this is what we found, this is where we’re at…came back, this is the diagnosis, it’s DCIS and that’s…it’s not…its pre-cancer, not cancer. I use the term DCIS and I say pre-cancer. Pre-invasive cancer or pre…yah, it’s usually pre-cancer. (03 surg onc)  so I tend to call it a pre-invasive cancer. (04 surg onc)  So the first thing that I usually do is frame it by telling the patient that she actually does not have breast cancer but that she has a pre-cancerous lesion in the breast. I do try to focus on that this is a pre-cancerous lesion, there’s no…I say that the cells do not have teeth, do not have legs, they cannot walk out of the duct or break free of the ducts and so they are completely contained. (06 surg onc)  I emphasize that it’s not a cancer. I emphasize that it is a pre-cancerous. I’m talking about pre-cancer so I’m not talking cancer. I emphasize that a lot because I don’t need any unnecessary anxiety from the patients that they have cancer. So I emphasize that if it’s a 100-percent cured for the most part with a treatment…which means it’s not really cancer. (07 surg onc)  it is a pre-cancerous change but the cells have not yet acquired the ability to become cancer cells and spread beyond the breast. So we think that if left untreated they might turn into cancer and therefore we take it very seriously but it’s not currently a cancerous condition…I explain that there’s a series of changes that normal breast cells have to undergo to become breast cancer cells that there are multiple stops along the way and that DCIS is one of those and kind of visually represent on the spectrum of those changes where that falls. (09 surg onc)  I basically say that this is a pre-cancerous thing, it’s not actually cancer but some of them will progress to cancer if we don’t do anything. (10 surg onc)  I think one of the key things that I do is I say…I say it’s a pre-cancer lesion rather than invasive cancer. And I really try and make that distinction to them, very, very clear because most of them come in thinking that they have breast cancer. So I use the word….so I use the word…the word that I use is pre-cancer. I say DCIS but then I say this is a pre-cancer and we think that it puts you at increased risk for developing in a cancer. (11 surg onc)  a pre-cursor for breast cancer but it is not truly cancer because it’s non-invasive(03 gen sx)  basically saying that you’ve got normal cells that sit and line the ducts of the breast and then some of those cells become abnormal with time but there’s few mutations in those cells and some of those mutations have characteristics of cancer and that is a DCIS or a ductal carcinoma in situ. It has cancer characteristics but the in situ part is just a fancy medical term for saying it doesn’t have the ability to spread yet. And so it stays within the ducts and without that ability to spread it’s not characterized as a full blown breast cancer. (04 gen sx)  I think usually much of the discussion revolves around actually terminology. They’re always confused by what DCIS is and I basically couch it as being a pre-cancer lesion or abnormality or growth of cells. (04 gen sx)  Usually pre-cancerous changes. But I do describe high risk evolution and high risk features that could suggest invasive disease or increased risk of invasive disease in the future. But I describe it as a pre-cancerous lesion. (07 gen sx)  I’m not using the terms stage. I’m gonna say, this is just a pre-cancerous lesion…but I’m not using stage, I’m gonna say, just pre-cancerous. (01 med onc)  Not cancer. Not cancer. This is not cancer. This is not cancer. Because there’s a lot of people are convinced that they have a malignant process and they don’t. (02 med onc)  so typically when patients see me they already know their diagnosis they may or may not remember it. They may not understand it. But I usually review by saying that they have a pre-cancer or not a true cancer; that the cells look like cancer cells but they don’t know how to escape the area that they grew in…I use the word pre-cancer. I talk about the cells, they might look like the same as cancer cells under the microscope but they haven’t yet learned how to escape the area where they grew in. So technically speaking there is no way that this cancer could spread. And that your survival is the same no matter what we do. (02 rad onc)  the first thing I discuss with them is what it means to have a DCIS while I usually explain that it’s sort of a pre-cancerous stage that doesn’t have the ability to involve the nodes or to spread into the other organs. (05 rad onc)  What I’m trying to do is you know usually when they come to me they really feel as if they have something that is life-threatening, they heard breast cancer and that’s how they feel. So that’s why I’m trying to use words for them to understand that it’s a serious condition that we’re trying to…we’re taking that very seriously for sure and that’s why they require surgery and they’re here at the cancer clinic. But on the other hand, I’m also trying to explain that this is not the typical breast cancer that they usually hear about in the media or when they read on the internet. usually these days I will say it’s sort of a pre-cancerous stage and I will sometimes explain that it’s the parallel with in situ cancer of the cervix just for them to understand that when they pap test they will find sometimes pre-cancerous changes and these also are taken seriously. (05 rad onc  Pre-cancer. Call it a pre-cancer. (06 rad onc)  DCIS is considered to be a pre-cancer lesion and then explain the difference between a pre-cancer lesion and the real cancer. (11 rad onc)  I call it like the pre-invasive for the…it’s an in situ cancer that hasn’t spread and may not result in any harm. (01 rad)  I usually start by saying that this isn’t cancer, it’s pre-cancer and just clarifying that especially because with the title, ductal carcinoma in situ; I feel like when people hear carcinoma they automatically think it’s…that they have cancer. So I just clarify with them that its pre-cancerous changes (02 RN)  We talk to it as being like a pre-cancer when we talk DCIS. It’s the terminology that we use. (03 RN)  it is described as a pre-cancer however I don’t like to fixate that that’s gonna be their final pathology. I usually try and leave it just as kind of a pre-cancer; cells that have the potential of becoming more active and becoming an invasive cancer if left alone. (06PN)  I find that most patients don’t really understand what DCIS is. I emphasize that it is a pre-malignant condition. I usually draw a diagram of the breast and how the different lobules fit in of 20/25 units and then I indicate on the diagram that the pathologies is confined to the ducts and differentiate the difference between that and an invasive carcinoma and how important that diagnosis is. (10 rad onc) |
|  | DCIS as a form of breast cancer/ early stage/ stage 0 | I go through the actual pathology report and tell them that this ductal carcinoma in situ, that it’s in early stage; that is really described as stage-0 (05 surg onc)  Probably the most commonly ways framed that I frame it is by saying and re-emphasizing that it’s breast cancer but I try to help the patient understand the distinction between non-invasive and invasive breast cancer. (05 gen sx)  until they’ve actually violated or penetrated through the base membrane it remains non-invasive breast cancer which I usually try to explain to the patients that it represents the best form of breast cancer if they were ever going to get problem of this nature. (05 gen sx)  I like to describe DCIS as a disease of cancer cells contained within the breast ducts and cancer cells that remain localized within that ductal system and have not broken through the ductal system into the …tissue into the lymphatics or into the blood vessels adjacent to the disease. (06 gen sx)  Some people talk about DCIS as being pre-cancer and patients often come in from the surgeons saying that you know oh no, I was told this was pre-cancer etc., but I don’t agree with that particular term and I…so I just, if that’s the case I just clarify them it is breast cancer but it’s you know very early contained within the ducts (04 rad onc)  I explain that it’s a breast neoplasm so you know basically it is a form of early malignant change in cells... I do tell people that generally this diagnosis is not a threat to somebody’s life but more often it’s a threat to the breast. (06 rad onc)  I explain that these are cancer cells, the individual cells look like cancer and that’s why it’s classified as a cancer but they aren’t demonstrating the ability to behave like a cancer….I talk about a true breast cancer being an invasive breast cancer and just clarify for them that invasive just means a real cancer (07 rad onc)  I usually start by explaining to the patient that the DCIS isn’t an invasive cancer. So which is stage-0 breast cancer; the DCIS itself is no threat to their health. The problem is that it represents a potential to develop into a real cancer and so the goal of treating it is to maintain their good health not that they’re currently sick. (07 rad onc)  I tend to say to them that it is described as stage-0 breast cancer and is pre-invasive but that it can recur and it can become invasive and therefore sometimes treatments such as surgery and radiation are required. I usually try to explain to them that it hasn’t gone beyond the milk ducts of the breast. (08 rad onc)  I usually tell her that it’s a form of cancer that doesn’t spread, although there is a chance of the cancer coming back locally and the….and then I’ll go on to explain the different risk factors for recurrence. it’s a very early form of breast cancer that does not spread. (12 rad onc)  I tell them that in situ means that the cancer hasn’t broken through the basic membrane of the duct that it’s in. And so it’s not considered an invasive cancer but it’s treated in a very similar manner. It is still considered to be a cancer and so it will require surgery if that is the diagnosis. (03 rad)  it’s a stage-0 cancer that you know that it’s not gone to the next step of learning how to spread. (04 RN)  I use the word baby breast cancer when I’m talking about DCIS. So it’s you know caught very early, stage-0, you know I use words like the cancer was all contained within the milk ducts; those types of terminologies. Sometimes it’s confusing because patients come and they say, well this isn’t really breast cancer, it’s a pre-cursor to breast cancer. And so I’m not sure if they’ve misunderstood or if it’s just the way that the diagnosis is being presented you know and then I usually say, well it is classed as a stage-0 and if left untreated there’s a good chance that it’ll become invasive. And so we look to do something before that happens. (05 RN) |
|  | Uses pre-cancer but acknowledges it is not ideal | so I tend to call it a pre-invasive cancer. And every time I say that, I hate saying that. I wish we had a different name for it. (04 surg onc)  I tend to call it pre-cancerous for patients understanding which is probably not the best most accurate description if you’re you know gonna be picky. But I think it’s probably the best for the lay person to understand. (10 surg onc)  it’s actually hard for me to explain that yes, it’s cancer but it’s not really because it doesn’t spread. And that the whole treatment is a little bit influx as to what it’s ideal. So that’s kind of how I sort of start out (01 gen sx)  I think they get way led into thinking this is something that it isn’t. This…I think the system does that and the fact that we call it cancer is part of the problem. (02 med onc) |
| Difficulty achieving patient understanding through discussion | Time spent distinguishing DCIS from breast cancer | mean I think you end up…I find that you end up spending a lot of time explaining to them the difference between DCIS and invasive breast cancer because they don’t necessarily understand that often when they come. They know they have breast cancer and that’s all they understand. the treatment that…to them has been you know the same as the treatment for someone with invasive breast cancer so. (04 med onc)  I would often draw a picture as to how these cancer cells are within the duct and therefore unlikely to travel by bloodstream to other areas in the body and how that relates to the risk of distant recurrence (03 med onc)  I actually use a picture. So we have this slide box that we have had in the clinic for as long as I’ve been there. That shows a picture of sort of the normal duct and then it shows DCIS then it shows invasive breast cancer. So I use that to show them the difference. so is it low grade, is it high grade, is it comedo type, what was the size of it, you know way in on whether or not I think they need to have further surgery which most times I don’t because that’s already been addressed before they come to me…So I use that because I find as a visual tool that’s helpful for them (04 med onc)  I show them you know and explain the difference between an in situ and an invasive cancer and the difference in the recommendations for treatment because of that. And then we talk about the clinical breast examination that they’re gonna have and the discussion with the surgeon. (01 RN)  I think it’s the medical literacy is a big issue, right? Like you know its bad terminology. DCIS is…because they don’t but is it cancer or is it not cancer. What do you mean it doesn’t have the capability of…like it’s just…you waste like 10-minutes trying to get them to understand that what in situ carcinoma is. So I think there is definitely a medical literacy component to it. You know it’s hard to get somebody to the operating room if they’re not really on board with what the diagnosis is. It’s hard to get them to buy into you know the treatment plan, that kind of thing. Yah, so that can be tricky. (04 gen sx) |
|  | Patients cannot comprehend diagnosis | And because I do…I do see my patients kind of at 6-month follow-up following surgery because it’s usually after they’ve had all the radiotherapy etc., they’ve met with all the other specialists. And when I see them again, sometimes patients are still asking me whether or not…you know they actually had cancer or not or whether it was DCIS or not. (04 gen sx)  I mean at the end of the day they couldn’t figure out really what they’re dealing with the breast cancer or this is a pre-cancerous lesion. This is the main question they have. And even when you see the patient after 6-weeks some follow-up after starting treatment they’re gonna go ask you the same question. You know they gonna ask you, doctor really I had a cancer? (01 med onc)  it’s like if I don’t think that they’re getting it so-to-speak, right? I only have like two or three different ways of explaining it and once I…you know if I get to my you know third way then …and they’re not really getting it and they’re you know…then that’s certainly uncomfortable (04 gen sx)  the confusing issue of like is this cancer or is it not. So I think that’s hard for patients to wrap their heads around. That we’ve given you this diagnosis but then we tell you that it’s pre-cancer but yet we’re recommending you to have treatment. So I think that’s probably the biggest thing, is like kind of understanding that concept of a in situ carcinoma which could turn into something…but technically is not at this moment in time. (02 rad onc)  I think the main barrier honestly for the most part is patients just having the capacity to understand the notion of a non-invasive breast cancer and the potential for it to develop into an invasive breast cancer and that it’s not currently a threat; that’s just some patients have a hard time wrapping their head around that notion. (07 rad onc)  DCIS is a complicated disease. It’s sometimes more complicated than invasive cancer. There are set guidelines and several trials which tell us what the results would be when a treatment is done or not done. A lot of that information isn’t…is not as much information is available for DCIS. And also it’s very confusing for the patient if you say, the options include no treatment versus mastectomy, tamoxifen, radiation; I mean that’s quite difficult for the patient’s actually to grasp and not only to the patient’s even to the physicians. So that’s the complexity of treatment is the third barrier. (09 rad onc)  Basically when I see the patient the problem is most of them, they are under the impression that they have an invasive breast cancer. So it takes my time to review the meaning of DCIS and let them know what really it means that you have DCIS…This is the first…it is very, very challenging. I don’t know from my…it’s very difficult to describe what it means that…DCIS, what it means invasion (01 med onc)  often their surgeon is sending them to us because they have been unable to calm the risk perception down. So they ask another colleague to talk more about risk perception. (02 med onc) |
|  |  |  |
| Justifying treatment recommendation with good prognosis |  | If the need a mastectomy they find it harder to understand why they need a mastectomy for pre-invasive disease. You know what I’m saying, it’s not that serious but you need a mastectomy. That can be a difficult discussion (03 surg onc)  the biggest challenge is potentially when I need to do a mastectomy for the DCIS and talking that through. (03 surg onc)  You know for a surgeon, the margin for DCIS when we do surgery has to be bigger than the margin for invasive disease. So it’s really backwards that DCIS you have to be more aggressive surgically to get a bigger margin. on the one had you’re trying to explain to the patient that it’s not really a cancer, so don’t worry. So I…but on the other hand you’re talking about the same treatment which you know surgery and radiation and probably endocrine therapy. And so really the emphasis becomes not on the less aggressive treatment, the emphasis becomes on don’t worry, it’s not gonna spread and there’s a tiny amount of chance that you have to balance it because you also have to give them a little heads up that there’s a tiny chance that it actually…maybe invasive disease already you just don’t know about. So you’re shifting the reason for why you are aggressive in surgery because the patients think that the reason why you’re treating it just as aggressively is because it’s cancer. (07 surg onc)  understanding that its stage-0 and they’re not gonna die and yet they need like a major operation. But more so is probably the challenge in getting them to agree…people with extensive DCIS, getting them to understand that they would benefit from a mastectomy when they have strage-0 disease. (08 surg onc) |
| Providing informational resources | Variable resources | I have my own personal website, which has a whole bunch of websites on it. So I kind of refer them to that to look at if they’re of that…wanting more information. (01 gen sx)  We provide them with a…basically an on-line link to the BC Cancer Agency website who has…has very good you know write-up of you know benign breast diseases, DCIS and breast cancer you know and it’s laid out nicely for patients. (04 gen sx)  if they ask questions about where they may look for additional information as well I’ll refer them to sites on the internet. I usually refer them to Up-to-Date. Up-to-Date patient part of it where people, general population can inquire and I find that one useful. (05 gen sx)  We talk about education on the web and the internet and staff…I have a kit for foundation based websites. We talk about the Canadian Breast Cancer Foundation, the Memorial Sloan-Ketering, University of Toronto website, Cancer Care Ontario; I try to tell them stay away from patient blogs and the New York Times. (07 gen sx)  I refer patients when they’re considering mastectomy and reconstruction. So I’ll refer them to the American Society of Plastic Surgery website because they have some good images and pictures. Otherwise not really, I don’t have a website that I’m sending patients too. (06 surg onc)  we have this big guide on breast and everything and anything you want to know about DCIS and invasive disease and so there’s websites listed in it. I can’t remember which ones but there’s …I think for support breast cancer actions and local support groups; then we have you know some different websites. I think the American Society of Breast Surgeons and things like that. (07 surg onc)  I haven’t had anybody ask. I don’t but that we do provide then with our internal information and there are some references there to publications that they can look for those that are that way minded. (08 surg onc)  If they look for it…I’m not sure who I’d send them too? If they’re young I send them to a…I send them to Young Adults Cancer Canada which is the YACC. But they specifically deal with people under 40 with all kinds of different cancer. So I sometimes send people there if they are under 40. Otherwise I’d sort of say you know you can contact the Canadian Cancer Society or somebody like that. But a lot of this is not done probably by me but through the navigators or the nurses in the clinic; if they have questions along those lines, like someone else ends up dealing with them more than me. (10 surg onc)  I use information from randomized trials because with DCIS there are many randomized trials; and very consistent information from the randomized trials. So I like to use that information…I encourage them to do if they want to look on the internet is to access the large cancer centre you know reputable information type thing. So you know in the U.S. you know Memorial Sloan Kettering or things like that and within Canada like our own websites good. And I say, you know Princess Margaret Hospital, Sunnybrook Hospital; those sorts of websites that give information for patients are reliable. (04 rad onc)  So I usually use those papers and hopefully the most recent papers to guide my discussion and my explanations and how I will explain what I recommend in terms of adjuvant treatments. So that’s my first tool, the recent literature (05 rad onc)  We have something that we provide to patients. There’s a video and there’s a brochure but it’s mostly about side effects of radiation or how you know they will experience the radiation treatments; so it’s a video about radiation. So it’s not truly about DCIS. When patients are asking for additional information usually I will direct them to the BCC website. In terms of other on-line resources I’m really reluctant to give them any idea because oh my goodness, it’s all over the place. And in terms of you know general publication website that are orientated for the general population I don’t know any that are you know reliable and good sources. So I usually ask them not to go too much on the internet. (05 rad onc)  I mean the printed materials that we have here in the cancer centre. There’s also Cancer Care Ontario has a practice guideline. So I will go over the practice guideline with individuals as well to you know to discuss that with them. (06 rad onc) |
| Facilitating assimilation, understanding and recall of information | Self-drawn images | I’ll often times draw a sketch or show diagrammatically you know how…how for instance DCIS hasn’t invaded through base membrane. So I draw a picture of what a milk duct looks like and show how cancer cells populate and multiply. (05 gen sx)  Lots of pictures. I draw or I’ll use…we have a pre-printed anatomical breast. So I do a lot of description of inter-luminal changes, cellular changes, so the cellular changes that happen. (07 gen sx)  I do individual sketch for all of my patients and then I give them that to take home with them….and it’s the same for everybody, and then I draw them a picture of the duct side the breast and then I show…abnormal clump of cells within the duct and I explain that that what’s DCIS does. And then as soon as those cells escape the ducts and start growing outside of the duct; so that’s when it becomes a breast cancer. And so that’s kind of my routine… when they go home and talk about it with family members it usually kind of jogs their memory (01 surg onc)  I draw a picture of what DCIS looks like versus invasive cancer. And so I’ll draw them a duct and a membrane…not a membrane but you know a duct and cancer is when the cells are outside and can’t…they’ve developed the ability to go outside it and that’s…it’s only when they develop the ability to go outside, can they just go somewhere else. And DCIS is not that. (03 surg onc)  Then I usually use drawings or I draw a duct and the line, the cells line in the duct and how do they grow into the lumen, it’s in sight if you go through the wall of the duct, it’s invasive. So I explain why this is called in situ. (05 surg onc)  So I basically draw a picture of duct you know make a little circle say here are the milk ducts and here are all these cells and they look like cancer cells if you just picked out an individual cell and looked at it. But they’re all contained in the duct and they haven’t gotten through the duct wall, the basic membrane and thus they can’t access the lymphatics and blood vessels theoretically. (10 surg onc)  I haven’t used any decision tools sorted of printed you know charts or anything like that…we don’t have any sort of pre-arranged information or video or any of that sort of information stuff. It’s just sort of me in the clinic doing a little back of the envelope description of what DCIS is. (10 surg onc)  I draw pictures of the breast and the ducts. And so I…one tool or strategy I use is drawing straight forward diagrams; number 1, number 2, using lay language. So the use of lay language as opposed to medical jargon I think is important. If you are trying to make the decision accessible to patients (01 rad onc)  I do my drawing, like my careful, my you know and I could use you know a standard picture of the breast or whatever but I find if I draw it out for the patient I can individualize it a little bit more to them; you know just in terms of if there was a positive margin or you know two little foci of DCIS or whatever. I can individualize my picture… I find if I draw a diagram and take the time to explain that to them they really understand it a lot better in terms of them going on and discussing why treatments recommended and you know about recurrences half the time being invasive, etc., etc. They understand that a lot better if I take the time to do that diagram and the explanation. (04 rad onc)  my kind of plan that I write on my own paper with what I think is their risk of relapse without radiation, with radiation, with or without endocrine. So I put numbers there but it’s not truly…it’s just…it’s more to guide the information I’ll provide to patients. It’s not something that they really access and there’s nothing else that I use apart from that. (05 rad onc)  actually I don’t really have any tools like diagrams or pamphlets or decision-aids or anything. I don’t…I just have me and my pen and paper and the patients sometimes I draw them a diagram of their breast; show them you now where the DCIS is. I describe how the surgery is done…take…normal tissue around the area and I sort of draw a picture of that. But I don’t have any formal tools beyond that. (07 rad onc)  I draw my own drawings and that’s it. Sometimes I show them the mammograms if they want to…especially if they’re dense breast or extensive macro-calcification if they chose to have a lumpectomy and if mastectomy is the better option for them then we recommend that (09 rad onc)  I always use my own diagram. I draw a separate…every time I sit with them draw…yah; I draw the outline of a breast. I find it more effective for me to use my own diagram. You know that’s where I can sort of isolate it to one ductal system. I can show them the branching. I can show them where the involvement is in relation to their breast. I can draw a diagram….a line to show the proximity of the resection margin to the actual tumor and explain why we need to give them additional treatment because it could be at multiple sites or they could be skip areas. And yah, I find that much more effective than using a printed diagram… I do, do is to give them the piece of paper…very important that they walk out with something. (10 rad onc)  I use a pen and a piece of paper. I draw a duct, a lobule, then I would indicate within the duct some abnormalities which would be my representation of DCIS and then I would draw that breaking through the wall of the duct and explaining that’s how you know once it breaks through the wall it would be an invasive component. I would then indicate the potential of it going to lymph nodes or not based on confined to the duct or not but I use just a very, very simple visual and that pretty much is it. I don’t use any big charts or fancy photos or anything like that. (11 rad onc)  sometimes I would kind of draw a little diagram of explaining how the DCIS cells look like cancer cells that only have not started to invade (12 rad onc)  I draw picture of the breast with calcifications or the abnormal area on it. And then I…and sometimes I`ll actually show them particularly in cases where it`s more extensive and I might recommend a mastectomy; sometimes showing them their mammogram so that they can see the extent of disease or will help them appreciate why I`m making that recommendation. (03 gen sx)  the diagram itself is on the patient teaching sheet that we hand-out but at the same time what we normally do is we draw it out in front of them because I think it sticks a bit better when you go step by step and draw it for them and then give that to them. But it is also on their teaching sheet; more of a professional version (03 RN)  usually the way we do it here is or what I would do is I would go back to the radiologist report and then I draw pictures of its calcifications, draw pictures and say you know small area calcifications, what quadrant of the breast they’re in and then just you know draw pictures saying, if we removed that area and we get a clean edge and they’re all removed then a lumpectomy is a reasonable option and then we usually add radiation afterwards. (04 RN) |
| **Recognizing and responding to emotions** | | Clinician responses |
| Issue | Theme |  |
| Clinicians emphasized good prognosis to reduce patient concerns | Positive message framing | I think the first thing that I like to do is to allay some fears. I spend a first bit of time telling them they’re not going to die; in plain language, so like putting in context that it’s not a full blown cancer. (02 surg onc)  I try and deescalate. I emphasize that it’s a non-invasive lesion and that there’s no threat of metastatic disease from a diagnosis of ductal carcinoma in situ and that there likelihood of dying as a result of this in long term data is almost zero. And I try and get away from its name, I hate its name. (04 surg onc)  I will walk out to the waiting room; call their name with the pathology report in my hand. I’ll say oh, Mrs. Craig, I hope the x-ray doctors didn’t hurt you too much on the biopsy. I’ve got a pretty good report for you, come on, let’s…as I’ve already started talking to them before I’m even in the room. That’s just…it’s probably our physical set-up, right? I got a little bit of a walk to do with them. I’ve already tried to deescalate the thing. Here’s your picture, it’s not a big area you know, that’s not gonna be very hard to remove at all. And then I go on from there. So really made an effort from the very beginning to get away from the idea that they have a horrible cancer. (04 surg onc)  I take the time…if I don’t explain then of course it’s not understood at all. But if I start by saying that from the first thing I say when I meet them, it sort of, it relaxes them, and it sets the tone for the rest of the consult. Before coming to see me they have all kinds of thinking about the fact that it is cancer, it could spread, all those things. And so as the first thing… is I try and engage that from the beginning. (07 surg onc)  I just wanted you to know you know just to emphasize from the beginning that you know this is early disease, it’s not considered cancer by half the world. That’s what I say, that half the world don’t even call this cancer and I’m one of them. so there’s no concern for that for spreading and I emphasize that it’s a 100-percent treatable and a 100-percent curable. (07 surg onc)  the first thing that I always stress to patients is that it is very early, very low risk breast cancer. so the first thing I stress is that it really…it has a very good prognosis and survival is extremely high, etc., etc. (04 rad onc)  I would typically explain that it is not invasive and therefore be not worried about distant metastases and therefore the prognosis is good and this is not affected life expectancy. (03 med onc)  I always say to people you know if we have a choice you would always choose in situ over an invasive breast cancer. (01 RN)  So I kind of try and turn it around to say this is actually a good thing because we’re catching this before it has a chance to even turn into anything really bad so. (02 RN)  If they’re anxious about the breast cancer and fearful, it’s harder for them to process information. So that’s why I always try to get them to be you know as calmed down as possible by talking about the prognosis first and then I think they hear some of the information better. (04 rad onc) |
| Providing tangible help to address emotions | Human resources (patient navigators) | Absolutely and these are high maintenance emotional needs patients. So having somebody that they can access after the consultation is over just alleviates a lot of their psychological consternation. (09 surg onc)  A nurse navigator or somebody who can listen to the questions and back the physician up when they’re finished is really helpful if you put various health units can afford it, it becomes down to money. But someone who can just sit there and walk them through and listen without them feeling pushed is really helpful (01 gen sx)  I think you know if we did have a kind of nurse navigator, nurse educator kind of person I think that would be really helpful. I think that person could…it would either they would…could do some of the things that I do, although I do think patients also like hearing it from the oncologist. But I think that you know it would…a lot of patients it’s helpful to have things reinforced (04 rad onc)  I do think that sort of better opportunities for patient education would be good because…I do find that women fear of breast cancer is often much, much higher than it needs to be with a diagnosis of DCIS. I think that, it would be nice to have more resources available other than just the oncologist or the nurse trying to kind of explain things. I do think it would be helpful to have more resources to help with that (04 rad onc)  I think a navigator is always helpful. I think that there are other situations where a navigator is more needed such as locally advanced breast cancer when there are more modalities of treatment being used and I think the treatment pathway is more difficult for patients in you know in those situations. So I think that a navigator could be helpful for DCIS but I don’t see it as the priority you know if we have limited resource and a limited number of navigators (08 rad onc)  You know I think that the navigator is very important. I’ve worked with a navigator at the hospital and it makes communication easy for the patient. And if they’ve got an issue they can sort of have it routed…re-routed to discuss with the doctor that they’re not happy to make the decision; whatever it is and we can go further than that. I have no problems with navigators at all and it could be useful. (10 rad onc)  they should just have somebody as we are outside the room, could be the navigator, saying, do you understand everything that’s going on? Are you happy to make a decision? Probably that kind of conversation would be better a few days later once they’ve got a chance to digest what was discussed by the surgeon and the radiation to that person; just giving them the opportunity to either reconsider. (10 rad onc)  a patient navigator, a nurse navigator can help particularly patients who will undergo reconstruction know potential downside of having reconstruction because I find that a lot of patients when they’re told that they have the breast cancer they…a lot of them decide to do reconstruction. But I don’t think they had…they were given time on what truly are involved in reconstruction. (12 rad onc) |
| **Managing Uncertainty** | | Clinician responses |
| Issue | Theme |  |
| Constructing and defining uncertainty | Uncertainty of progression rarely addressed / addressed using vague terms | this is the kind of a red flag. It means basically you would be at risk of…your risk of getting the same kind of the pre-cancerous lesion or invasive breast cancer higher than the general population at your age. So for me, this is a red flag that needs much more attention and maybe you benefit from it. (01 med onc)  So I claim it as it’s a benign condition associated with the risk of subsequent malignancy. (02 med onc)  It’s not really invasive in a breast cancer but put you at risk of invasive breast cancer in the same breast or the other breast, contralateral breast. (01 med onc)  it’s not actually cancer but some of them will progress to cancer if we don’t do anything. Which ones that happens to is hard to figure out and thus most people end up getting treated for DCIS to make it…you know to radiate it; to get it gone and fully treated and away. The other component to that is that most of the time people come with core biopsies of DCIS and to a surgeon and some of those people will actually be upstaged when we get the whole lesion out to actual cancer which they have to understand that too. So I say that the diagnosis may change once we sort of got the whole lesion figured out. (10 surg onc)  it’s a lesion that if left alone could turn into cancer in the years to come and that’s the reason to remove it. (02 surg onc)  We don’t know if its gonna go onto develop cancer but we usually recommend treating it because we don’t know if it will. (03 surg onc) |
|  | Uncertainty of progression justification for treatment | we need to take them out now because I explain sometimes that changes with time if it’s left in situ or sometimes the biopsy can miss actual cancer cells that have already made those changes. So we need to remove that (04 gen sx)  so like putting in context that it’s not a full blown cancer although it’s just a biopsy so it could be cancerous on final biopsy. And it’s a lesion that if left alone could turn into cancer in the years to come and that’s the reason to remove it. (02 surg onc)  we don’t have to look at anything else in terms of you know staging and I say that sometimes when we do, do surgery we will find some invasive disease, I prepare them for that possibility. (03 surg onc)  we have to also rule out an invasive component because even though the imaging and the biopsy that we have show only the DCIS is always a possibility or find it invasive. And I explain that to the patient as well. (05 surg onc)  there’s always a possibility of finding an invasive component later, therefore it’s…not my need to be the one after. I also explain to them that they have to wait for the final pathology to again assess that it’s only DCIS; the extent of the disease, the grading, the hormone receptives if needed and usually it takes 2-weeks. So I tell them that I’ll see them 2-weeks after surgery. (05 surg onc)  So the first thing that I usually do is frame it by telling the patient that she actually does not have breast cancer but that she has a pre-cancerous lesion in the breast. And I spend a fair bit of time discussing what that means and that these changes could if left alone possibly but not definitely become a breast cancer but also that these changes could already harbor a breast cancer that perhaps we’re just not seeing on the biopsy (06 surg onc) |
| **Making Decisions** | | Clinician responses |
| Issue | Theme |  |
| Preparing for the decision and deliberation/ making a choice | Provides all options and discusses with patient | Well we talk about the multidisciplinary approach with regards to surgery, radiation, plus or minus chemo prevention. We talk as from a surgical point of view; we talk about partial mastectomy versus mastectomy and the different approaches to that. (07 gen sx)  we talk about what surgical options the patients have based on the size of the tumor and where the tumor is located in the breast. So I give them what their options are in terms of like mastectomies versus breast conservation. [If] they have any prior knowledge from whether it’s family members or friends or whatever and kind of if they’ve done any reading and what kind of choices they already preconceived had prior to the visit. And then ask whatever questions they have and then usually come up with a plan at that point; it’s about what our surgical management is going to be whether it’s a lumpectomy or whether it’s a mastectomy. And then we talk about kind of any adjuvant treatments that would be required afterwards just to kind of touch upon it. (01 surg onc)  And then depending on the size of the area we’ll be talking about lumpectomy, radiation versus mastectomy…of course, if it’s too large for …lumpectomy then we have to talk about mastectomy with or without reconstruction. (03 surg onc)  I make sure I know before I go in with the patient whether or not it is amenable to do breast conserving surgery. make sure they have no contraindications of radiation in the sense that having had previous radiation or connected tissue disease and as long as all of those things are favorable then I can present to them the options. But I don’t want to present options that are inappropriate. (08 surg onc)  when I determine that they’re able to do either then I can present the option of breast conserving surgery with radiation versus mastectomy. (08 surg onc)  the 3-end points that are of interest is avoidance…what’s the risk of local recurrence of the breast of any sort. What’s the risk of an invasive local recurrence? And what’s the chance that they might die of breast cancer. then the options for treatment would be number one, nothing further. Number two would be mastectomy. Number three would be radiation of the breast. Number four would be radiation with tamoxifen or endocrine therapy anyway. So those would be main four things that we need to discuss. And then the risk varies with various things like the patient’s age and the size and the grade and how close the margins were and that sort of thing (01 rad onc)  when the patient comes to me I discuss the option of radiation versus no radiation versus systemic treatment with tamoxifen; that’s the main purpose of my discussion. But I also mention that mastectomy is still an option if the patient changes her mind with regards to radiation, tamoxifen or the option of surveillance. So when they see us they actually have four options, right? Mastectomy that’s completion mastectomy, radiation, no treatment at all, surveillance versus tamoxifen with no radiation (09 rad onc) |
|  | Recommends/ advocates for one option | somewhere in that discussion I will sort of say, this is the whole range of treatment options but because you’re in “x” category I would think you know bilateral mastectomy is too radical; just doing a lumpectomy is fine (01 gen sx)  So typically I sit down with the women, I say you know because this is a relatively smaller area in relation to your breast you have the option of just removing the area. Or we have the option of removing the entire breast. I’ll often then say in this setting because typically it is in the setting of DCIS. I say that a mastectomy in this setting is an aggressive option; it is not a wrong option. It’s a personal preference but it would be aggressive here. There’s no cancer benefit to removing more normal breast tissue. I often explain it to them in terms of like a colon cancer. And I say you know gawd forbid your doctor told you, you had a cancer in your intestine you wouldn’t say you know doctor, take my entire intestine. You would say you know take the parts that you need and we’re gonna watch the rest of it. So similarly here, the name of the game is get rid of the changes and prevent future changes from occurring but you don’t live longer because we removed more normal breast tissue. And that’s how I usually start the conversation. (06 surg onc)  if I have a women that… in that discussion expresses an interest for a mastectomy despite this being amenable to a lumpectomy, I will often bring them back for another discussion. I think there’s a little bit of shock in the initial diagnosis and most women and I often ask them at the beginning, you know do you have a gut feeling? some women will come in thinking, okay if this is a breast cancer diagnosis, I’m having a mastectomy. You know in other women come in with the you know thought of you know I want to preserve my breast if possible. So if they have a preconceived thought for a mastectomy then often just speaking with them and going through the lack of benefit in terms of survival …for a mastectomy over lumpectomy makes them re-think things. But there’s often the desire for mastectomy is an unrealistic understanding of your risk of recurrence. So by reframing their expectation often they come back and say okay, you know I’ve thought about it and if it is safe then I’m willing…then I would have a lumpectomy. Or if they still want a mastectomy and then we go ahead with that. (06 surg onc)  usually if you can just tell them, no look here’s the …here are the exact issues with how long you’re going to live if you do this, that or the other. And there’s no influence of survival doing the other, plus it’s just…surgery, plus it’s going to delay your treatment for the other one in case you know you get any kind of wound infection. Anyway, I can usually at least get them to just accept to get the things treated that they have now and then they can worry about the other one later if it’s still on their mind. (02 gen sx)  I focus on that the next step is a small step, it’s very likely it’s the only surgery they will ever need but the information we learn from that small step will help inform the further decisions. So it’s not, not been ruled out but I emphasize we’re gonna do this little thing first and then we’ll use that to see where we should go. (04 surg onc)  if someone says, you know Doctor, should I have my breast off? I say you know, let’s slow down here. We don’t even have the whole area looked at by the pathologist yet, right? So I’m pretty reluctant to go down that route. And if they’re really leaning towards that, you know I emphasize you know well let’s do this small step first. I keep coming back to that strategy of we’re gonna do this little thing first and that doesn’t mean you can’t have a mastectomy. But it means you’re gonna have more information to help make that decision. And invariably I find time after time the woman who may have really been enthusiastic about a mastectomy when she gets a reassuring report of the entire thing removed and again, the same thing. The report is printed; the pertinent features are underlined, highlighted. I make little notes on the side. They keep that, right? And they look and say, oh, oh, yah this does look pretty good. (04 surg onc)  I go to the treatment choices and I tell them that the current recommendation is to do surgery; to do a lumpectomy, remove the area. I stress the importance of having clear margins or removing it completely. And then I explain to them that we probably need additional treatment with radiotherapy for local control to prevent them from coming back in the breast itself. And there might be a role for systemic treatment; tamoxafin to reduce the risk in the same breast or the opposite breast. (05 surg onc)  I never said no completely to the mastectomy. I explain to the patient that it’s not indicated, it’s not needed but it’s their decision in the end. And I always kind of post-pone the decision because I…and I tell the patient this is not the time to make that decision, you’re scared, a new diagnosis; think about it, take your time, I don’t want you to do a more aggressive surgery and then regret having done it because it’s something that you really did not need. So I think it’s important to let the patients think and sleep over the diagnosis, think more about it. And then usually patients are reasonable and they accept the fact that they do need a more aggressive surgery especially for DCIS (05 surg onc)  then I go ahead and I talk about the different surgical options to remove it emphasizing that because it’s not really a cancer that we don’t have to be…you know we have to make sure that the patient understands why we often don’t check the lymph nodes and….but I try and emphasize if possible breast conserving surgery. And I end with the idea that we’re gonna book the surgery and that after the surgery we will confirm that it was all DCIS and nothing else and then we’ll send them to the oncologist after. So I end with a plan for treatment. (07 surg onc)  I generally would not present a mastectomy and lumpectomy as equal options for DCIS if anyone was a candidate for breast conserving surgery. I would certainly encourage that and recommend it and typically that requires wire localization because these don’t usually present as a mass. if somebody has a large area of DCIS then I certainly to talk about mastectomy and reconstruction…that some women choose to have a mastectomy for this condition I certainly make it very clear that for that woman that is not necessary and it could be completely addressed without a mastectomy with a much lesser surgery, less invasive, fewer complications, etc,, and then I would encourage that. (09 surg onc) |
|  | Does not always inform patient of options | if the area looks readily ressectable I don’t even mention the word mastectomy. I just say we’re gonna, will get this area out and the pathologist will go through the whole thing and then we’ll learn more about it and we’ll bring you back and then we can discuss it further. I tell them think of the next step as almost one of your tests. And we’re gonna get more information and then that will guide us on from there. Maybe you’ll…you might need…you might consider a precautionary course of radiation if it’s…depending on the features of it. (04 surg onc)  then I tell them that the treatment is typically to cut it out and often to follow that up with radiation and then we talk about hormone therapy as well. (09 surg onc)  For the smaller DCIS’s I sort of lay it out and say you‘re gonna have lumpectomy and almost…radiation with that or you can have a mastectomy instead if they ask, aim them towards breast conservation especially for small lesion because they’re small and they probably don’t need to have a big operation for it. (10 surg onc) |
| **Enabling patient self-management** | | Clinician responses |
| Issue | Theme |  |
| Preparing, implementing and assisting | Limited DCIS specific educational resources | I don’t think there’s anything that’s specific to DCIS. I haven’t looked at it in a while. A lot of it is kind of it is around the surgery and what to expect at the time of surgery and if they need a wire localization procedure. What that involves and that kind of stuff. I don’t know if there’s a lot of kind of pathophysiology information of the breast cancer and the DCIS. (02 surg onc)  we do have a pamphlet that we’ll give out through the Cancer Centre. It’s just a very general overview, so it’s not specific. (07 gen sx)  then there’s a big …there’s a sort of a workbook for…which is generic for all patients with breast cancer that’s available through the Cancer Centre. And the nurses go through that and all of them…all patients go home with one of those. (01 rad onc)  We give everyone a package of information, the purple loop-in kit which still floats around from I think the Canadian Cancer Society or somebody. So we give them that but we don’t necessarily go through it in clinic. (10 surg onc)  In general it’s breast cancer radiation type of this. I don’t think we have anything specific for DCIS unless they’re participating in a clinical trial where you have the material to give to them. I do not have any specific material specific to DCIS to give to the patients. (09 rad onc)  So I don’t think I have any specific-like tools in terms of…yah, any information that like given to patient other than the tamoxifen hand-out which isn’t really specific to DCIS; just a patient hand-out on tamoxifen and its side effects. Yah, I don’t know that I use any specific aids. (02 rad onc)  we do have some general booklets about radiation in our department and in our facility here. We have some other general literature that’s given to all our breast patients and I have an information sheet that I have myself, with…that’s very specific to radiation and the breast. And so I…that has not so much to do with DCIS but has to do with the radiation side effects on the breast; so that’s very specific side effects and complications of radiation to the breast and I provide that to the patients as well. (11 rad onc) |
|  | Limited psychological resources | it would be nice if we have a support group for them but I don’t know if I have that kind of time to arrange that. DCIS is only about you know 20-percent of all patients that we see and we’re so busy dealing with the invasive disease that you know I know sometimes there’s less resources and less emphasis on DCIS because people think it’s not cancer. And sometimes I find that the DCIS patients are even more anxious than the invasive people; only because it’s like they think they almost had cancer and that somehow more anxiety provoking than actually having it. And so I think these people probably do need some support. (07 surg onc)  I think sufficient psychosocial support for women who need it and kind of sufficient education materials, access to information. I think a social worker who’s familiar with breast cancer patient with the context of DCIS would certainly be helpful who can then also make decisions or triage if more support would be needed. (03 med onc)  a psychologist who can talk to them because DCIS is not the same as say an invasive ovarian cancer or head and neck cancer where …or advanced lung cancer. People…women with DCIS should not associate their cancer with the death, right? So some sort of a psychosocial support. (09 rad onc)  they often have higher needs than some of the invasive breast cancers in regards to their supports. They’re just more high needs patients. They just tend to demand more and more…they struggle more with trying to make decisions, those kinds of things. Like when given the option of the radiation or given the option of hormone therapy they struggle more with making those informed choices. (11 rad onc) |

| **Other** | | Clinician response |
| --- | --- | --- |
| Issue | Theme |  |
| Recommended interventions | Disinterest in additional tools | I have a lot already. Like I said, we…and patients will find their own. We do you know suggest a local website that then will allow access to other on-line resources and our brochures is paper-based. So I don’t think there is a lot out there. I haven’t really had patients say, you know I really don’t know what I’m doing or what this is about. So I don’t know. I don’t know that there really is. (03 surg onc)  I mean because there are clinical guidelines do exist. We have provincial guidelines for management of DCIS. And they do actually do cover a lot of that. I don’t know that there’s a need for something extra specifically on communication around DCIS. (03 surg onc)  I actually don’t think there is. I can’t think of where it would be helpful for me or haven’t had any patient’s articulate sort of a disappointment or a need. I don’t know, I don’t see patients as feeling as lost as invasive patients in terms of wishing they had the support group or. So I don’t know. (08 surg onc)  that I think our communication is generally very good and patients exhibit good understanding at the end and have a chance to ask all their questions and I’ve not had anybody expressed dissatisfaction with the process as it is right now…I find the conceptual notion that these are not yet cancer cells therefore they are still in the breast and do not have…they’ve not gone anywhere else in your body; is sufficient explanation for people. (09 surg onc)  so much is available I don’t think I need it in my practice. I think so much of it is available now on social media and there’s lots of support groups; they’re very active in my area. So I find that patients are really well informed and they’re well informed before they see me most of the time. I mean videos are always good, years before, probably we relied on them more in clinics and stuff. But I think now people can go through on-line social media, YouTube at their leisure can go through some pretty accurate video stuff. (05 gen sx)  I’ve got a pretty refined process that I probably even if something was really great and available I’d probably you know… not a tool to start using it. I’d be factious a bit, but probably I over 30-years evolved approach that I find usually works. And patients leave smiling and seen a lot less stress than when the conversation starts. So that’s usually a good sign. So I probably wouldn’t personally find additional tools development helpful (01 rad onc)  The communication issue is something they you know this would be a model type case where if you were teaching a communication course to medical trainees, you could say okay let’s take the cases of DCIS where there’s a lot of options and how…and try and model that it’s appropriate. I think that would be a valuable thing. (01 rad onc)  know we have lots of hand-outs. A patient library place is full with hand-outs and the number of times…and we’ve given patients books of things about…and a number of times the people read it and understand it I think is disappointing… So there’s nothing to substitute for the … the doctor and the patient (10 rad onc)  You know I don’t really know that I need any more tools. So I don’t really know what else we can do besides just spend that quality time. (01 RN)  I feel like we’re really well-rounded here because we really…I can’t even think of anything. Like I…I think we do as much as we can. (02 RN) |
